# Arterial catheter-related infection of 2,949 catheters

**DOI:** 10.1186/cc4930

**Published:** 2006-05-24

**Authors:** Leonardo Lorente, Ruth Santacreu, María M Martín, Alejandro Jiménez, María L Mora

**Affiliations:** 1 Department of Intensive Care, Hospital Universitario de Canarias, Santa Cruz de Tenerife, Spain; 2 Research Unit, Hospital Universitario de Canarias, Santa Cruz de Tenerife, Spain

## Abstract

**Introduction:**

Which particular arterial catheter site is associated with a higher risk of infection remains controversial. The Centers for Disease Control and Prevention guidelines of 1996 and the latest guidelines of 2002 make no recommendation about which site or sites minimize the risk of catheter-related infection. The objective of the present study was to analyze the incidence of catheter-related local infection (CRLI) and catheter-related bloodstream infection (CRBSI) of arterial catheters according to different access sites.

**Methods:**

We performed a prospective observational study of all consecutive patients admitted to the 24 bed medical and surgical intensive care unit of a 650 bed university hospital during three years (1 May 2000 to 30 April 2003).

**Results:**

A total of 2,018 patients was admitted to the intensive care unit during the study period. The number of arterial catheters, the number of days of arterial catheterization, the number of CRLIs and the number of CRBSIs were as follows: total, 2,949, 17,057, 20 and 10; radial, 2,088, 12,007, 9 and 3; brachial, 112, 649, 0 and 0; dorsalis pedis, 131, 754, 0 and 0; and femoral, 618, 3,647, 11 and 7. The CRLI incidence was significantly higher for femoral access (3.02/1,000 catheter-days) than for radial access (0.75/1,000 catheter-days) (odds ratio, 1.5; 95% confidence interval, 1.10–2.13; *P *= 0.01). The CRBSI incidence was significantly higher for femoral access (1.92/1,000 catheter-days) than for radial access (0.25/1,000 catheter-days) (odds ratio, 1.9; 95% confidence interval, 1.15–3.41; *P *= 0.009).

**Conclusion:**

Our results suggest that a femoral site increases the risk of arterial catheter-related infection.

## Introduction

Arterial catheterization is a frequent procedure in intensive care units (ICUs). In the European Prevalence of Infection in Intensive Care study, for example, 44% of critically ill patients underwent arterial catheterization [1]. Arterial catheters are used when frequent arterial blood sampling or continual monitoring of systemic arterial pressure is considered necessary.

Intravascular catheters may cause different complications, including infection. The interest in catheter-related infection research lies in the attributable mortality [2-5] and the attributable costs [6-9] it represents.

In a previous study developed by our team [10], we analyzed catheter-related local infection (CRLI) and catheter-related bloodstream infection (CRBSI) of 1,231 arterial catheters (radial, brachial, dorsalis pedis and femoral) and 1,608 central venous catheters (subclavian, jugular and femoral) reported for each site. We only found a significantly higher incidence of CRLI in femoral and jugular venous access as compared with subclavian access. In that study, there were no significant differences in the incidence of CRLI or CRBSI between the different arterial catheter sites.

Although there are many studies on arterial catheter-related infection [11-24], we have found only two studies that presented the information completely for each arterial access, as in our study [11,12]. The number of femoral arterial catheters used, however, was only 12 cases and no case, respectively; the number of arterial catheters used (340 and 70, respectively) was also lower than in our current study (2,949 arterial catheters, of which 618 were inserted at the femoral site).

Whether a particular arterial catheter site is associated with higher risk of infection remains controversial. Significant differences in the incidence of CRLI [24] and CRBSI [11] between different sites have not been found, although in these studies the number of arterial catheters (186 and 340, respectively) was lower than in our current study (2,949 arterial catheters). In the Centers for Disease Control and Prevention (CDC) guidelines of 1996 [25], and in the latest guidelines of 2002 [26], there is no recommendation about which arterial catheter insertion site should be used to minimize the risk of infection.

In a second study, we increased the number of arterial catheters to 2,949 and increased the number of central venous catheters to 2,595 [27], in order to increase the probability of finding other significant differences.

The objective of the present study was to compare the incidence of CRLI and CRBSI of arterial catheters according to different access sites.

## Materials and methods

A three year prospective study involved all patients admitted to the 24 bed ICU of Hospital Universitario de Canarias (Tenerife, Spain), between 1 May 2000 and 30 April 2003. The study was approved by the institutional review board.

The catheters used were not antimicrobial coated, but were radiopaque polyurethane catheters (Arrow, Reading, PA, USA). The placement and maintenance of catheters were performed according to the following protocol. The catheters were inserted by physicians with the following sterile-barrier precautions: use of large sterile drapes around the insertion site, surgical antiseptic hand wash, and sterile gown, gloves, mask and cap.

The skin insertion site was first disinfected with 10% povidone-iodine and anesthetized with 2% mepivacaine. The catheters were inserted percutaneously using the Seldinger technique and were fixed to the skin with 2–0 silk suture. After line insertion the area surrounding the catheter was cleaned with a sterile gauze soaked with povidone-iodine, and a dry sterile gauze occlusive dressing then covered the site. No topical antimicrobial ointment was applied to insertion sites.

The percutaneous entry sites were examined for the presence of local inflammation and purulence, and were cared for daily in the same manner by the ICU nurse assigned to the patient. Catheter dressings were changed every 24 hours, or sooner at the discretion of the nurse if the dressing was contaminated. The connecting lines were changed every 48 hours and disposable transducer components were replaced every 96 hours.

The percutaneous entry sites were also examined daily by the ICU nurse assigned to the patient to avoid accidental catheter removals [28] and to minimize infection risk associated with reinsertion of the catheter.

The decision to remove the catheter was made by the patient's physician. Catheters were removed when they were no longer needed or if a systemic or local complication occurred. Arterial catheters were routinely replaced every seven days. The insertion site for the new catheter was changed when the catheters were replaced. All catheter tips removed were routinely cultured.

The catheters were removed using a sterile technique by the ICU nurse. The distal five cm segment of the catheters was cut with sterile scissors, placed in a sterile transport tube and cultured using the semiquantitative method described by Maki and colleagues [29].

The following data were collected: age, sex, diagnosis, Acute Physiology and Chronic Health Evaluation (APACHE) II score, ICU admission and discharge dates, catheter site, catheter insertion and removal dates, cause of catheter removal, and development of CRLI and CRBSI. We studied the following four groups of arterial catheter sites: radial, femoral, dorsalis pedis and brachial.

Catheter-related infection was defined according to catheter-tip colonization, CRLI and CRBSI. We considered catheter-tip colonization as a significant growth of a microorganism (>15 colony-forming units) from the catheter tip. CRLI was considered as any sign of local infection (induration, erythema, heat, pain, purulent drainage) and catheter-tip colonization. CRBSI was considered a positive blood culture obtained from a peripheral vein, and signs of systemic infection (fever, chills, and/or hypotension), with no apparent source of bacteremia except for the catheter, and catheter-tip colonization with the same organism.

The age, APACHE II score and total duration with the index catheter are expressed as the mean ± standard deviation. The total duration of ICU stay is reported as the median (interquartile range). Categorical variables are expressed as percentages.

The CRLI and the CRBSI are reported as follows: the percentage of catheters that developed CRLI and the number of CRLIs per 1,000 catheter-days, and the percentage of catheters that developed CRBSIs and the number of CRBSIs per 1,000 catheter-days.

The comparisons of the arterial catheter sites on age, the APACHE II score, the total duration with the index catheter and the total duration of ICU stay were carried out with Kruskall-Wallis test for independent samples. The chi-square test was used for comparing proportions between the different arterial catheter sites on the diagnosis groups, the order of catheter insertion and diabetes mellitus. The comparisons of the incidence, per catheter-days, of CRLI and CRBSI between the different arterial catheter sites were performed using four Poisson regression analyses.

Eight models were constructed for adjusting by the total duration of ICU stay, the diagnosis group, the order of catheter insertion and sex. The arterial catheter site was the main independent variable. The rates of CRLI and CRBSI were introduced as dependent variables.

An *a posteriori *comparison was carried out among the four arterial catheter sites. Statistical analyses were performed with SPSS 12.0.1 (SPSS Inc., Chicago, IL, USA) and LogXact 4.1 (Cyrus Mehta and Nitin Patel, Cambridge, MA, USA).

The magnitude of the effects is expressed as the odds ratio and 95% confidence interval. *P *< 0.05 was considered statistically significant. Inferences are based on exact *P *values (Poisson analysis) and asymptotic *P *values (comparisons of arterial catheter sites on age, APACHE II score, total duration with the index catheter and the total duration of ICU stay).

## Results

During the study period 2,018 patients were admitted to the ICU, of whom 1,243 (61.60%) were male. The mean age was 56.85 ± 19.52 years, the mean APACHE II score was 13.81 ± 5.97 and the mean duration of ICU stay was 8.86 ± 13.18 days; 262 (12.98%) patients died. Admission diagnoses were as follows: 907 (44.95%) heart surgery, 278 (13.78%) trauma, 257 (12.71%) neurologic, 234 (11.60%) cardiac, 199 (9.86%) respiratory, 91 (4.51%) patients digestive and 52 (2.58%) intoxication.

A total of 1,775 (87.96%) patients underwent arterial catheterization. The number of arterial catheters and the days of arterial catheterization were as follows: total, 2,949 and 17,057; radial, 2,088 and 12,007; brachial, 112 and 649; dorsalis pedis, 131 and 754; and femoral, 618 and 3,647. The incidences of CRLI and CRBSI were 1.17/1,000 catheter-days and 0.59/1,000 catheter-days, respectively (Table 1).

In the univariate analysis, no differences were found between the different arterial catheter sites on diabetes (*P *= 0.49), age (*P *= 0.72), APACHE II score (*P *= 0.90), total duration with the index catheter (*P *= 0.49) or sex (*P = *0.14) (Table 2). Only sex was included on the multivariable analysis because its *P *value was less than 0.20.

However, find differences between the arterial catheter sites on the total duration of ICU stay (*P *< 0.001), the diagnosis group (*P <*0.001) and the order of catheter insertion (*P *< 0.001) (Table 2). These variables were included in the multivariable analysis because the *P *value was less than 0.05.

As the number of cases with positive events of CRLI and CRBSI was low (20 and 10, respectively), a full multivariate model including the total duration of ICU stay, the diagnosis group, the order of catheter insertion, sex and the arterial catheter site was not possible. The only option for multivariate analysis was to construct eight partial models, including the arterial catheter site as the main independent variable and one of the confounder variables (total duration of ICU stay, diagnosis group, order of catheter insertion or sex) as independent variables; the rates of CRLI and CRBSI were introduced as dependent variables in each partial model.

The arterial catheter site showed statistical significance in the eight partial models. We report only results for two partial models adjusted for the total duration of ICU stay. As shown in Table 3, the CRLI incidence was higher for femoral access (3.02/1,000 catheter-days) than for radial access (0.75/1,000 catheter-days) (odds ratio, 1.5; 95% confidence interval, 1.10–2.13; *P *= 0.01). Table 4 shows that the CRBSI incidence was higher for femoral access (1.92/1,000 catheter-days) than for radial access (0.25/1,000 catheter-days) (odds ratio, 1.9; 95% confidence interval, 1.15–3.41; *P *= 0.009).

A total of 10 microorganisms were responsible for the 10 CRBSIs: six coagulase-negative staphylococci, three *Escherichia coli *and one *Staphylococcus aureus*.

## Discussion

In the present study we found that the femoral site had a significantly higher incidence of CRBSI and CRLI than the radial arterial site.

The literature contains two studies that analyzed catheter-related infection in detail [11,12]. The number of femoral arterial catheters used (only 12 cases and no cases, respectively) and the number of arterial catheters used (340 and 70, respectively) however, were lower than in our study (2,949 arterial catheters, of which 618 were inserted at the femoral site).

We have found two studies reporting that 0.2% [13] and 3.8% [11] of arterial catheters developed CRLI; our percentage of arterial catheters developing CRLI (0.68%) was near to this lower rate. We have found one study that reported a CRLI incidence of 15.64 infections/1,000 catheter-days [11]; our incidence of CRLI was lower (1.17/1,000 catheterdays), probably because our CRLI definition was more restrictive and required the presence of catheter-tip colonization.

According to the literature, 0–13% of arterial catheters develop CRBSI [11-20] and the incidence of CRBSI ranges from 0 to 12/1,000 catheter-days [11,12,14-17,21]. Our findings were near to this lower limit (0.34% arterial catheters developed CRBSI, and the CRBSI incidence was 0.59/1,000 catheter-days).

Which particular arterial catheterization site is associated with higher risk of infection remains controversial. In relation to CRLI, in one study [24] there were no significant differences between femoral and radial sites (25% versus 21%) – although in the study by Thomas and colleagues the number of arterial catheters (186 arterial catheters) was lower than in our study (2,949 arterial catheters). In relation to CRBSI, there were no significant differences between the two access sites in one study [11], although in the study by Furfaro and colleagues the number of total arterial catheters and the number of femoral access sites (340 and 12, respectively) were lower than in our study (2,949 and 618, respectively). In the present study, the femoral site was associated with significantly more CRLIs and CRBSIs than the radial site. The higher incidence of catheter-related infection in femoral sites than radial sites is probably due to the higher density of local skin flora on the groin area [18].

We routinely changed arterial catheters every seven days, based on four reasons. In two studies, arterial catheterization longer than 4 days was associated with a higher risk of catheter-related infection [11,17]. Second, in other studies arterial catheters were routinely changed every 3–8 days [15,22]. In one study it was suggested that the low incidence of catheter-tip colonization and no CRBSI may be due to the fact that most arterial catheters were removed within four days [16]. Finally, the CDC guidelines of 1996 recommended replacing arterial catheters no more frequently than every four days, but no recommendation for the maximum duration was made [25]. The current CDC guidelines of 2002, however, recommend not routinely replacing arterial catheters to prevent catheter-related infections [26].

The limitations of this study were as follows. First, different insertion sites were not randomly assigned. No randomized trials, however, have compared infection rates for arterial catheters placed in the four different sites. Only in the study by Thomas and colleagues [24] were the patients randomly assigned to undergo arterial catheter insertion at the femoral or the radial site.

Second, as the number of cases with positive events of CRLI and CRBSI was low, a full multivariate model including the total duration of ICU stay, the diagnosis group, the order of catheter insertion, sex and the arterial catheter site was not possible. The only option for multivariate analysis was to construct eight partial models, including arterial catheter site and a confounder variable as independent variables in each partial model, and all partial models showed a significant effect of the arterial catheter site on CRLI and CRBSI.

The third limitation was the CRLI definition used. Our definition of CRLI included both criteria (any sign of local infection and a positive semiquantitative culture of the catheter tip). This definition is one of the possible criteria of arterial infection according to the 1988 CDC criteria [30]. In the CDC guidelines of 1996 [25] and 2002 [26], the CRLI definition did not require a positive culture of the insertion site, and the following aspects of CRLI were distinguished: exit site infection, pocket infection and tunnel infection.

## Conclusion

In the CDC guidelines of 1996, and in the latest guidelines of 2002, there is no recommendation about arterial catheter site insertion to minimize infection risk. The logical inference from our results is that avoidance of the femoral site may help minimize the risk of arterial catheter-related infection.

## Key messages

• 	In order to minimize catheter-related infection, it is necessary to monitor its incidence and to implement preventive measures.

• 	We found that the femoral site had a significantly higher incidence of CRLI and CRBSI than the radial arterial site.

• 	The logical inference from our results is that avoidance of the femoral access site may help minimize the risk of arterial catheter-related infection.

## Abbreviations

APACHE = Acute Physiology and Chronic Health Evaluation; CDC = Centers for Disease Control and Prevention; CRBSI = catheter-related bloodstream infection; CRLI = catheter-related local infection; ICU = intensive care unit.

## Competing interests

The authors declare that they have no competing interests.

## Authors' contributions

LL was responsible for conception and design of the study, data collection, and analysis and interpretation of results. RS and MMM performed data collection. AJ performed statistical analysis and interpretation of the results. MLM was responsible for conception and design of the study and interpretation of the results. All authors approved the final version of the manuscript to be published.

## Figures and Tables

**Table 1:** Description of catheter-related local infection (CRLI) and catheter-related bloodstream infection (CRBSI) of arterial catheters inserted at various sites

Arterial catheter site	Number of arterial catheters	Days of arterial catheters	Number of CRLI	Incidence of CRLI^a^	Arterial catheters with CRLI (%)	Number of CRBSI	Incidence of CRBSI^a^	Arterial catheters with CRBSI (%)
Radial	2,088	12,007	9	0.75	0.43	3	0.25	0.14
Brachial	112	649	0	0	0	0	0	0
Dorsalis pedis	131	754	0	0	0	0	0	0
Femoral	618	3,647	11	3.02	1.78	7	1.92	1.13
Total	2,949	17,057	20	1.17	0.68	10	0.59	0.34

^a^Incidence defined as the number of infections per 1,000 catheter-days.

**Table 2:** Baseline characteristics of the patients and arterial catheters

	Arterial catheter site				
	
	Radial (*n *= 2,088)	Brachial (*n *= 112)	Dorsalis pedis (*n *= 131)	Femoral (*n *= 618)	*P *value
Age (years) (mean ± standard deviation)	56.75 ± 17.1	57.87 ± 16.8	58.20 ± 17.2	56.74 ± 17.2	0.72
Sex (female) (*n *(%))	876 (42.0)	58 (51.8)	51 (38.9)	251 (40.6)	0.14
Diabetes mellitus (*n *(%))	695 (33.3)	32 (28.6)	49 (37.4)	199 (32.2)	0.49
APACHE II score (mean ± standard deviation)	13.83 ± 5.0	14.03 ± 5.2	13.55 ± 4.8	13.83 ± 5.0	0.90
Diagnosis group (*n *(%))					
Coronary artery bypass grafting	589 (28.2)	24 (21.4)	26 (19.8)	42 (6.8)	<0.001
Valve surgery	456 (21.8)	20 (17.9)	20 (15.3)	46 (7.4)	<0.001
Mixed surgery (coronary and valve)	66 (3.2)	3 (2.7)	4 (3.1)	4 (0.6)	0.007
Other cardiac surgeries	11 (0.5)	1 (0.9)	1 (0.8)	1 (0.2)	0.56
Cardiac	199 (9.5)	16 (14.3)	19 (14.5)	114 (18.4)	<0.001
Respiratory	175 (8.4)	11 (9.8)	15 (11.5)	88 (14.2)	<0.001
Digestive	48 (2.3)	3 (2.7)	4 (3.1)	34 (5.5)	<0.001
Neurological	240 (11.5)	15 (13.4)	19 (14.5)	123 (19.9)	<0.001
Traumatology	266 (12.7)	17 (15.2)	20 (15.3)	139 (22.5)	<0.001
Intoxication	38 (1.8)	2 (1.8)	3 (2.3)	27 (4.4)	0.004
Total duration of ICU stay (days) (median (interquartile range))	3 (3)	9 (8)	9 (9)	10 (10)	<0.001
Order of catheter insertion (*n *(%))					
First	1464 (70.1)	34 (30.4)	25 (19.1)	152 (24.6)	<0.001
Second	422 (20.2)	56 (50.0)	75 (57.3)	306 (49.5)	<0.001
Third	142 (6.8)	18 (16.1)	23 (17.6)	124 (20.0)	<0.001
Fourth	50 (2.4)	4 (3.6)	8 (6.1)	31 (5.0)	0.002
Fifth	10 (0.5)	0 (0)	0 (0)	5 (0.8)	0.49
Total duration with the index catheter (days) (mean ± standard deviation)	5.75 ± 2.2	5.79 ± 2.2	5.75 ± 2.2	5.90 ± 2.1	0.46

APACHE, Acute Physiology and Chronic Health Evaluation; ICU, intensive care unit.

**Table 3:** Comparisons of catheter-related local infection incidence (number of infections per 1000 catheter-days) between different arterial sites adjusted for the total duration of intensive care unit (ICU) stay

	Incidence	Odds ratio (95% confidence interval)	*P *value
Arterial catheter site			0.009
Femoral versus radial	3.02 versus 0.75	1.5 (1.10–2.13)	0.01
Femoral versus brachial	3.02 versus 0	1.6 (0.67-infinite)	0.32
Femoral versus dorsalis pedis	3.02 versus 0	3.2 (0.51-infinite)	0.26
Dorsalis pedis versus brachial	0 versus 0	1.0 (0.00-infinite)	0.99
Dorsalis pedis versus radial	0 versus 0.75	1.1 (0.00–2.84)	0.99
Brachial versus radial	0 versus 0.75	1.5 (0.00–9.35)	0.99
Total duration of ICU stay		1.03 (1.01–1.05)	0.01

**Table 4:** Comparisons of catheter-related bloodstream infection incidence (number of infections per 1000 catheter-days) between different arterial sites adjusted for the total duration of intensive care unit (ICU) stay

	Incidence	Odds ratio (95% confidence interval)	*P *value
Arterial catheter site			0.007
Femoral versus radial	1.92 versus 0.25	1.9 (1.15–3.41)	0.009
Femoral versus brachial	1.92 versus 0	1.3 (0.51-infinite)	0.63
Femoral versus dorsalis pedis	1.92 versus 0	2.0 (0.30-infinite)	0.54
Dorsalis pedis versus brachial	0 versus 0	1.0 (0.00-infinite)	0.99
Dorsalis pedis versus radial	0 versus 0.25	2.0 (0.00–6.22)	0.99
Brachial versus radial	0 versus 0.25	4.8 (0.00–44.7)	0.99
Total duration of ICU stay		1.03 (0.99–1.06)	0.06
